# The rising burden of obesity-linked cancers in Saudi Arabia: a public health review

**DOI:** 10.1097/MS9.0000000000004775

**Published:** 2026-02-20

**Authors:** Mohsin Yahya Murshid, Esam Mohammad Murshid, Nedal Bukhari, Mohammad Alfayez, Tareq Salah, Yasir Bahadur

**Affiliations:** a Department of Surgery, Hera General Hospital, Makkah, Saudi Arabia; b Department of Oncology, Prince Sultan Medical Military City (PSMMC), Ministry of Defense Health Services (MODHS), Riyadh, Saudi Arabia; c Faculty of Medicine, Assiut University, Assiut, Egypt; d Faculty of Medicine, King Abdulaziz University, Jeddah, Saudi Arabia

**Keywords:** body mass index (BMI), cancer, cancer prevention, noncommunicable diseases, obesity, obesity-related cancers, public health, Saudi Arabia

## Abstract

Obesity is a growing global health crisis with wide-ranging effects beyond metabolic and cardiovascular disorders. A substantial body of international evidence has linked excess adiposity to increased risk for various cancers. In Saudi Arabia, where obesity rates are among the highest globally, the relationship between obesity and cancer remains insufficiently characterized. This narrative review synthesizes global and regional literature on obesity-associated cancers, with a specific focus on the epidemiologic and research landscape in Saudi Arabia. A targeted search of PubMed, Google Scholar, and official databases from the World Health Organization, International Agency for Research on Cancer, and GLOBOCAN was conducted for English-language articles published between 1990 and 2023. The review highlights several cancers – postmenopausal breast, colorectal, liver, endometrial, kidney, and pancreatic – with convincing or probable associations with obesity. Alarmingly, many of these cancers also rank among the most common and fatal malignancies in Saudi Arabia, as shown in GLOBOCAN 2022 data. However, local research quantifying this link is limited. National cancer registries lack obesity-related variables, and longitudinal cohort studies are scarce. These gaps hinder the ability to implement evidence-based, obesity-targeted cancer prevention strategies. Given the convergence of high obesity prevalence and rising incidence of obesity-related cancers, urgent attention is needed to integrate obesity metrics into national cancer surveillance. Strategic investments in population-based cohort studies and registry reform are essential to inform tailored public health interventions and reduce the burden of obesity-related cancers in the Kingdom.

## Introduction

Obesity is a widespread health challenge on a global scale, having a negative impact on individual well-being and healthcare costs. Several variables have been identified as potential contributors to this phenomenon, including sustained economic development, shifts in lifestyle patterns, and a notable decrease in family size since the late 1970s^[1]^. The rise in overweight and obesity rates worldwide seems to be primarily influenced by internal factors such as economic growth and urbanization^[2]^. At a regional level, the prevalence of obesity is highest among men residing in high-income Western countries, with a rate of 27.2%. Conversely, women in Central Asia, the Middle East, and North Africa exhibit the highest obesity prevalence, with a rate of 31.4%^[3]^. According to a comprehensive survey conducted across Saudi Arabia, it was determined that the prevalence of obesity, when adjusted for national weightage, stood at 24.7%. Furthermore, a substantial correlation was observed between obesity and a range of health issues, hence underscoring the imperative for targeted interventions to address the issue of obesity within the country^[4]^.

**HIGHLIGHTS**

- Obesity is a well-established risk factor for multiple cancer types, including breast, colorectal, liver, endometrial, and kidney cancers.
- Saudi Arabia has one of the highest obesity prevalence rates globally, with over one-third of adults classified as obese.
- Many of the most common cancers in Saudi Arabia are also obesity-linked, exacerbating the national cancer burden.
- National cancer registries currently lack obesity-related data, limiting the ability to analyze trends and implement targeted prevention strategies.
- The review identifies critical gaps in epidemiological research and proposes recommendations for registry reform, cohort studies, and region-specific interventions.

The economic impact of obesity is significant in both developed and developing nations, necessitating the immediate implementation of public health interventions to prevent it. Additionally, there is a pressing need for global agreement on standardized approaches to estimating the financial implications of obesity, which should encompass the evaluation of obesity-related illnesses^[5]^. Given the seemingly unavoidable nature of modernization on a global scale, it is reasonable to anticipate a worldwide expansion of the overweight and obesity epidemic in the coming years^[6]^. The deleterious effects of obesity on health are widely acknowledged and encompass prevalent ailments such as diabetes, cardiovascular disorders, cancer, osteoarthritis, major depressive disorder, unfavorable pregnancy outcomes, and infertility^[7]^. Over the course of the last two decades, there has been a growing body of evidence indicating that obesity is a significant risk factor for specific forms of cancer^[8]^. Furthermore, an increasing body of research indicates that obesity may have a negative impact on outcomes in specific types of cancer^[9]^. Cancer has conventionally been predominantly perceived as a condition characterized by uncontrolled cell growth, although contemporary perspectives propose its classification as a metabolic problem as well^[10,11]^. The proposed recommendation was principally grounded in the concept of cancer-related alterations in metabolism at the tissue (tumor) level^[11]^. In the present context, it is noteworthy to observe that recent years have revealed a probable convergence in the potential mechanisms that could establish a connection between obesity, cancer, and cardiovascular-metabolic disorders, such as type 2 diabetes mellitus (T2DM) or coronary heart disease^[12]^.

This review provides a synthesis of global evidence linking obesity to the incidence of various cancer types and emphasizes the relevance of these associations within the Saudi Arabian context. Despite the country’s high prevalence of obesity, there remains a significant gap in national research addressing its impact on cancer risk. The primary objective of this review is to identify key research priorities that can guide future national studies and surveillance initiatives aimed at understanding and mitigating obesity-related cancer burden in Saudi Arabia. By outlining existing evidence and highlighting gaps, this work seeks to inform researchers and public health stakeholders about areas requiring further investigation rather than to propose policy recommendations. This review follows the TITAN 2025 checklist for transparent AI reporting^[13]^.

## Methods

This narrative review was conducted to synthesize global and regional evidence regarding the relationship between obesity and cancer, with particular emphasis on data relevant to the Saudi Arabian population. The objective was to examine epidemiologic trends, mechanistic pathways, and research gaps that hold significance for national health policy and clinical practice.

A comprehensive literature search was performed using multiple databases, including PubMed, Google Scholar, and ScienceDirect, as well as institutional sources such as the World Health Organization (WHO) and the International Agency for Research on Cancer (IARC). The search incorporated combinations of relevant keywords and MeSH terms, including “obesity,” “body mass index,” “cancer,” “obesity-related cancers,” “Saudi Arabia,” “cancer incidence,” “GLOBOCAN,” “public health,” and “non-communicable diseases.” Publications in English between January 1990 and December 2023 were considered for inclusion. Additional studies were identified through manual screening of reference lists from relevant articles and reviews.

Studies were included if they were peer-reviewed and explored the association between obesity and cancer incidence, mortality, or mechanistic links. Reports from national and international health organizations such as the WHO, IARC, and the World Cancer Research Fund (WCRF) were also reviewed, particularly those providing regional or global cancer surveillance data. Studies focusing solely on pediatric populations, underweight or malnourished cohorts, or experimental animal models were excluded.

The review prioritized systematic reviews, meta-analyses, large epidemiological studies, and registry-based datasets, such as the Global Burden of Disease and GLOBOCAN reports. Eligible studies were examined for their methodology, population characteristics, cancer type, and key findings. Given the heterogeneity in study designs and outcome measures, data were narratively synthesized rather than pooled quantitatively.

To enhance contextual relevance, epidemiologic data from Saudi Arabia were integrated where available, alongside global evidence. Quantitative results were derived from recent large-scale meta-analyses published between 2000 and 2024, particularly for insulin-sensitive malignancies such as liver, pancreas, and endometrial cancers, as well as for colorectal and breast cancers. The review followed the general structure of the TITAN 2025 guidelines for narrative reviews. Because this study was based solely on publicly available data and did not involve human participants, ethical approval was not required

## Global burden of obesity

Obesity has increased sharply worldwide over the past few decades. Between 1975 and 2014, global age-standardized obesity prevalence rose from 3.2% to 10.8% in men and from 6.4% to 14.9% in women^[3]^. Since 1980, the rates of overweight and obesity have more than doubled, and today they affect nearly one-third of the global population^[14]^. The rise in obesity spans all age-groups and both sexes, but it is especially high among women and the elderly^[15]^. However, the rates vary widely between regions. Obesity remains more prevalent in high-income and some middle-income countries. In nations like the United States, Sweden, Norway, Japan, and Australia, obesity trends have recently plateaued^[16]^. In contrast, many low- and middle-income countries are still experiencing a rise – especially in urban areas. For example, China has seen a major increase in age-adjusted obesity rates over the last two decades^[17,18]^.

Among children under 5, the trends are also worrying. Overweight rates have risen by 24% in Africa since 2000. In Asia, nearly half of the children under 5 were either overweight or obese by 2019^[19]^. The WHO reports a dual burden in sub-Saharan Africa. Adult obesity now coexists with childhood undernutrition, including stunting and wasting^[20]^. A pooled analysis by the NCD Risk Factor Collaboration assessed 128.9 million people aged ≥5 years from 1975 to 2016. It found major increases in body mass index (BMI), particularly in youth^[21]^. BMI rose by 0.32 kg/m^2^ per decade in girls and 0.40 kg/m^2^ in boys. By 2016, average BMIs were nearly equal – 18.6 for girls and 18.5 for boys. For adults, mean BMIs reached 24.8 in women and 24.5 in men. Polynesia and Micronesia had the highest regional BMIs, while South Asia and East Africa had the lowest^[21]^. By 2016, childhood obesity prevalence had climbed to 5.6% in girls and 7.8% in boys. Southern Africa showed the steepest rise. Adult obesity also surged. In 2016, 390 million women and 281 million men were classified as obese. The largest increases were seen in East Asia, the Middle East, North Africa, South Asia, and high-income English-speaking countries^[3,22]^.

**Figure 1.**
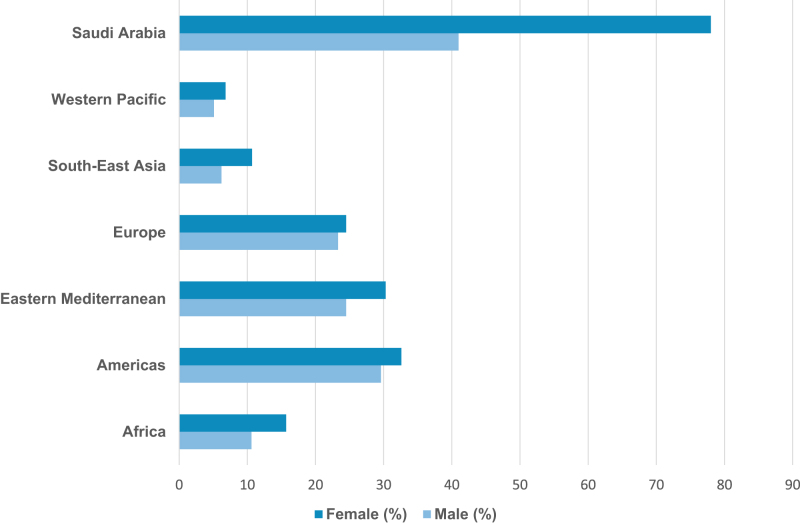
Adult obesity prevalence by WHO region (2020–2022), with Saudi Arabia shown for comparison. Source: WHO GHO; World Obesity Atlas; Althumiri *et al*^[32]^.

These trends call for urgent global action. Multinational and region-specific strategies are needed to prevent obesity across all age-groups. A global comparison of obesity prevalence across WHO regions, shown in Figure 1, highlights the Eastern Mediterranean Region – and particularly Saudi Arabia – as one of the most affected globally.

## Prevalence of obesity in Saudi Arabia

Prevalence of obesity in Saudi Arabia exceeds the global averages. National data estimate a 35% obesity prevalence, compared to 13% worldwide, and as a result, Saudi Arabia experiences a much higher obesity-attributable mortality rate^[23]^. Obesity is responsible for 18% of all deaths in the Kingdom, versus 8% globally, and the mortality rate is 116.7 per 100 000 population, compared to 60 per 100 000 worldwide^[22]^. Many large epidemiological studies have assessed obesity across Saudi regions. Eight studies, each with more than 10 000 participants, reported obesity prevalence rates as high as 35.6%^[24–31]^. These findings align with the Saudi Health Interview Survey (2013), which reported obesity in 24.1% of men and 33.5% of women^[31]^. Regional differences are also significant. A nationwide study by Althumiri *et al* found the highest obesity prevalence in the Eastern Region; in this region, 29.4% of adults had a BMI >30 kg/m^2^^[32]^.

**Figure 2.**
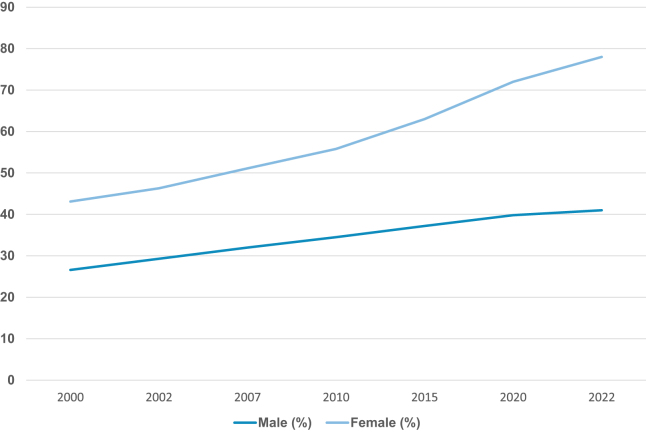
Trend of obesity prevalence in Saudi Arabia (2000–2022): obesity prevalence in Saudi Arabia has risen sharply between 2000 and 2022, with a particularly steep increase among women.

Obesity prevalence rises with age and is consistently higher among women. One national study found that women were twice as likely to be obese compared to men^[33]^. The Saudi-PURE study confirmed this gender gap; it showed that while men had more Class I obesity (BMI 31–35 kg/m^2^), women had far more Class II and III obesity (BMI >35 kg/m^2^), with 26.1% of women falling into this category, compared to 14.5% of men^[24–26,29–31,33–35]^. The increased prevalence rates also raise concerns regarding the increasing burden of noncommunicable diseases in the nation, such as cancer, diabetes, and cardiovascular diseases, all of which are intricately linked to obesity. Figure 2 depicts the significant increase in obesity prevalence in Saudi Arabia from 2000 to 2022, with a much greater impact on women.

## Biological mechanisms linking obesity to cancer

The biological link between obesity and cancer is supported by both epidemiological and molecular studies. Excess body fat causes systemic and cellular changes that promote cancer initiation, progression, and poor outcomes. These changes involve chronic inflammation, hormonal imbalance, metabolic disruption, and immune dysfunction. One of the well-established mechanisms is chronic low-grade inflammation. In obesity, adipose tissue becomes infiltrated by immune cells, especially macrophages. These cells release pro-inflammatory cytokines such as interleukin-6 (IL-6), tumor necrosis factor-alpha (TNF-α), and C-reactive protein, which contribute to DNA damage, angiogenesis, and cell proliferation, all of which are key processes in the development of cancer^[7,8]^. Insulin resistance and hyperinsulinemia, both common in obesity, also play a major role. Elevated insulin and insulin-like growth factor-1 (IGF-1) levels activate growth-promoting pathways like PI3K/Akt/mTOR. These pathways reduce apoptosis and enhance tumor survival and growth^[9,12]^. They are associated with several cancer types, including breast, colon, pancreas, and endometrium.

**Figure 3.**
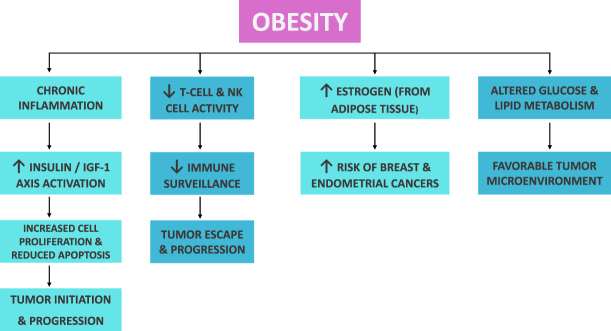
Mechanisms linking obesity to cancer development.

Hormonal changes are particularly important in certain hormone-sensitive cancers. In postmenopausal women, fatty tissue becomes the main source of estrogen production, which leads to higher circulating estrogen levels. The resulting hyperestrogenism increases the risk of postmenopausal breast and endometrial cancers by stimulating estrogen receptor-mediated cell proliferation^[8,9]^. Obesity also affects the tumor microenvironment. Expansion of fatty tissue causes it to become hypoxic, which leads to fibrosis and increased oxidative stress, supporting tumor progression. This condition also negatively impacts immune surveillance. T-cell and natural killer cell function is impaired, reducing the body’s ability to detect and eliminate cancer cells^[9,10]^. Recent studies have highlighted how obesity and cancers are involved in altered glucose and lipid metabolism, supporting the idea of cancer as a metabolic disease^[10,11]^. Together, these mechanisms explain why obesity is now seen not just as a comorbidity but as a direct cause of cancer. Understanding these pathways may help develop targeted prevention and treatment strategies for individuals at higher risk of cancer due to obesity. Figure 3 summarizes the key biological mechanisms that link obesity to cancer development.

## Cancers linked to obesity

The idea that obesity contributes to the development of cancer was first proposed in the early 20th century. In the 1930s, researchers suggested that excessive food intake, often seen in patients with cancer, might be linked to tumor growth^[36]^. However, early observations lacked solid evidence, and it was not until the late 1990s and early 2000s that the link became clearer. In 1997, key reports from the WCRF and the American Institute for Cancer Research drew global attention to the relationship between diet, body fat, physical activity, and cancer risk^[37]^. This paper was followed by a 2002 monograph from the IARC and a WHO report in 2003, both emphasizing cancer prevention through weight control and healthy lifestyles^[38,39]^. These reports laid the groundwork for a scientific consensus. Obesity is now strongly associated with a higher risk of several cancers, including colorectal, postmenopausal breast, endometrial, renal cell carcinoma, and esophageal adenocarcinoma^[39]^.

A major prospective study by Calle *et al* in 2003 added strong support to this evidence. They demonstrated that obesity increases overall cancer mortality in both men and women^[40]^. Their findings also linked obesity to higher death rates from breast, colon, endometrial, kidney, esophageal, liver, gallbladder, pancreatic, cervical, ovarian, and prostate cancers. Non-Hodgkin lymphoma, multiple myeloma, and leukemia were also included. In 2007, WCRF published a second global report. It reviewed more than 7000 studies and reinforced the link between body fatness and cancer^[41]^. The Continuous Update Project (CUP) now provides regular updates. Based on CUP findings, there is convincing evidence linking obesity to cancers of the colon, breast (postmenopausal), endometrium, kidney, esophagus (adenocarcinoma), liver, and pancreas^[41,42]^. Obesity is also considered a probable cause of other cancers, including ovarian, advanced prostate, gallbladder, and gastric cardia cancers^[43–46]^. In 2016, the IARC Working Group expanded this list, confirming additional links to thyroid cancer, meningioma, and multiple myeloma^[47]^. These findings combined support a strong and growing body of evidence. Obesity is a modifiable risk factor for numerous types of cancer. Reducing obesity at the population level could help lower both cancer incidence and mortality.

## The obesity–diabetes–cancer triad

Obesity and T2DM are metabolically intertwined disorders that together form a biologic continuum often referred to as *diabesity*. In this continuum, obesity induces insulin resistance, prompting compensatory hyperinsulinemia that gradually progresses to overt diabetes. Within the cancer framework, T2DM functions as both a confounder – because it coexists with obesity – and a mediator, lying on the causal pathway that links obesity to cancer development. Failure to account for diabetes in analytic models can therefore obscure or exaggerate the true magnitude of obesity-related cancer risk. Globally, robust epidemiologic evidence demonstrates that T2DM independently increases the risk of several malignancies. Large cohort and meta-analytic studies published between 2018 and 2024 report pooled relative risks (RRs) or hazard ratios (HRs) ranging from 1.2 to 2.5, depending on tumor site^[48–62]^. The excess risk is particularly pronounced for insulin-sensitive tissues—liver (RR 1.5–2.5)^[48–53,63–72]^, pancreas (RR 1.5–2.0)^[73–77]^, and endometrium (RR 1.6–2.2)^[54–61]^. More modest elevations are observed for colorectal cancer (RR ≈ 1.2–1.3) 145–149, 150–151 and postmenopausal breast cancer (RR ≈ 1.1–1.3)^[62,78–81]^. These associations are consistent across geographic regions and persist after controlling for major confounders such as age, adiposity, smoking, and physical inactivity. Collectively, the combined influence of obesity and T2DM likely accounts for up to one-third of the global cancer burden^[82–85]^.

The public-health implications of this triad are especially critical in Saudi Arabia, where the prevalence of diabetes and obesity are among the highest worldwide. National surveys and meta-analyses estimate an adult T2DM prevalence of 23–27%, exceeding 30% in older adults^[86–88]^. A pooled estimate of pre-diabetes around 24% further underscores the expanding at-risk population^[89]^. Local studies also report that diabetic and obese Saudi patients have significantly higher odds of colorectal cancer diagnosis and recurrence^[90]^. Together, these data illustrate a pressing need to integrate diabetes variables into both epidemiologic and registry-based cancer research in the Kingdom.

Diabetes influences cancer risk through several shared biological mechanisms that overlap with, but also amplify, those induced by obesity. First, chronic hyperinsulinemia in insulin-resistant states activates insulin and hybrid IGF-1 receptors, stimulating the PI3K–AKT–mTOR and MAPK signaling cascades that promote cell proliferation and inhibit apoptosis^[91,92]^. Elevated insulin also suppresses IGF-binding proteins 1 and 2, increasing free IGF-1 levels and further enhancing mitogenic activity.

Second, persistent hyperglycemia drives the Warburg effect shift toward aerobic glycolysis that provides rapidly dividing cells with abundant energy and biosynthetic precursors. Excess glucose promotes generation of reactive oxygen species (ROS) and advanced glycation end-products, leading to oxidative DNA damage, mutagenesis, and genomic instability^[93,94]^. Third, both obesity and T2DM sustain a state of low-grade chronic inflammation. Hypertrophic adipose tissue becomes infiltrated by macrophages and T cells that secrete pro-inflammatory cytokines such as TNF-α, IL-6, and IL-1β, while adipokine balance shifts toward higher leptin and lower adiponectin levels^[95,96]^. These changes activate NF-κB and STAT3 pathways that support tumor initiation, angiogenesis, and resistance to apoptosis^[97]^.

Fourth, insulin resistance is associated with lipotoxicity – accumulation of free fatty acids and ectopic lipids in organs such as the liver and pancreas – causing endoplasmic-reticulum and mitochondrial stress, ROS overproduction, and additional DNA damage. These mechanisms contribute to the high incidence of non-alcoholic fatty liver diseaseand non-alcoholic steatohepatitis observed among diabetic patients, both of which are precursors to hepatocellular carcinoma^[98,99]^.

Finally, prolonged metabolic and oxidative stress induces epigenetic remodeling, including DNA-methylation changes, histone modifications, and altered microRNA expression, all of which can silence tumor-suppressor genes or activate oncogenes^[100]^.

Taken together, these overlapping processes form a reinforcing feedback loop: obesity initiates insulin resistance and inflammation; diabetes amplifies hyperinsulinemia, hyperglycemia, and oxidative stress; and the convergence of these abnormalities accelerates carcinogenesis. In practical terms, T2DM mediates a substantial proportion of the cancer risk originally attributed to obesity alone. Meta-analytic mediation models estimate that up to 20–40% of the obesity-associated risk for liver and pancreatic cancers may be transmitted through diabetic mechanisms^[82,101,102]^.

In the Saudi context, where nearly one in four adults has diabetes and two in three are overweight or obese, these interactions have significant implications for prevention and policy. National registries and cohort studies should routinely record BMI, waist circumference, fasting glucose, HbA1c, and insulin levels to enable accurate adjustment for diabetes status and glycemic control. Analyses should ideally include stratified comparisons between diabetic and non-diabetic populations and employ causal mediation techniques to quantify indirect effects. At a population level, integrated strategies targeting weight reduction, early diabetes detection, and glycemic optimization could synergistically reduce the national burden of obesity-linked cancers. Table 1 summarizes cancer risk estimates patients with T2DM.

**Table 1:** Cancer risk estimates in patients with type 2 diabetes mellitus

Cancer site	Summary risk estimate	Statistic type	Direction/Interpretation
Liver (HCC)	RR 1.5–2.5 (SRR ≈ 1.94; 95% CI 1.66–2.27)	Meta-analysis/Cohort	↑ Strong association
Pancreas	RR 1.5–2.0 (long-term); RR 7.9 (new-onset <1 yr)	Meta-analysis	↑ Very strong, bidirectional
Endometrium	RR 1.6–2.2 (SRR 1.89; 95% CI 1.46–2.45)	Meta-analysis	↑ Strong, independent of BMI
Colorectum	RR 1.2–1.3 (HR 1.26; 95% CI 1.18–1.33)	Cohort/Pooled	↑ Moderate
Postmenopausal Breast	RR 1.1–1.3 (SRR 1.25; 95% CI 1.20–1.29)	Meta-analysis	↑ Mild-to-moderate
Thyroid	RR 1.3–1.4 (95% CI 1.22–1.44)	Meta-analysis	↑ Moderate
Kidney	RR 1.3–1.4 (95% CI 1.10–1.72)	Meta-analysis	↑ Moderate
Bladder/Urothelial	RR 1.3–1.4 (95% CI 1.17–1.56)	Meta-analysis	↑ Modest
Gastric	RR 1.1–1.2 (SRR 1.14; 95% CI 1.04–1.26)	Meta-analysis	↑ Small
Esophagus (EAC)	SRR 1.3 (95% CI 1.12–1.50); EAC ≈ 2.1 (95% CI 1.01–4.46)	Meta-analysis	↑ Small overall; strong for EAC
Oral cavity	–	Observational	↑ Qualitative
Lung	RR 1.03 (95% CI 0.94–1.13)	Meta-analysis	↔ No independent effect
Ovary (EOC)	RR 1.17–1.19 (95% CI 1.02–1.34)	Meta-analysis	↑ Weak
Cervix	–	Cohort	↑ Possible
Prostate	RR 0.85 (95% CI 0.82–0.88)	Cohort	↓ Inverse (incidence only)
Testis	RR 0.90 (95% CI 0.66–1.22)	Cohort	↔ No effect
Melanoma	RR 0.81 (ASR 83→65 ♀; 115→93 ♂)	Cohort	↓ Inverse
Hematologic (NHL, MM, leukemia)	RR 1.1–1.3 (95% CI 1.04–1.32)	Meta-analysis	↑ Small

EAC, esophageal adenocarcinoma; EOC, epithelial ovarian cancer; HCC, hepatocellular carcinoma; NHL, non-Hodgkin Lymphoma; MM, multiple myeloma; RR, relative risk; SRR, summary relative risk; HR,= hazard ratio; ↑ increased risk; ↓ decreased risk; ↔ no association.

Source: Pliszka, M.; Szablewski, L. Associations between Diabetes Mellitus and Selected Cancers. *Int. J. Mol. Sci.* 2024, *25*, 7476^[103]^.

## Common obesity-linked cancers and the Saudi cancer burden

**Figure 4.**
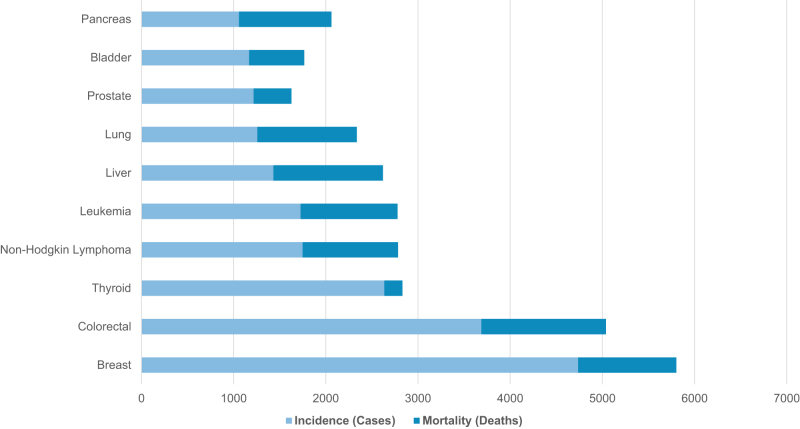
Incidence and mortality for the top 10 cancers in Saudi Arabia (2022). Obesity-linked cancers are highlighted in red. Source: GLOBOCAN 2022.

**Figure 5.**
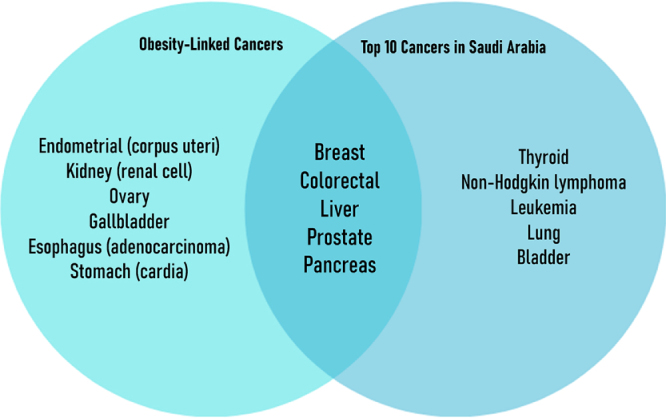
Overlap between obesity-linked cancers and the top 10 most common cancers in Saudi Arabia. Five cancers (e.g., breast, colorectal, liver) fall in both categories, highlighting preventable disease burden. Source: GLOBOCAN 2022; WCRF/IARC.

Several cancers which are strongly associated with obesity also rank among the most common malignancies in Saudi Arabia, raising important public health concerns. Obesity is a recognized risk factor for breast (postmenopausal), colorectal (colon and rectum), liver, corpus uteri (endometrial), kidney, pancreas, and ovary cancers^[42,43,45–47]^. In 2022, breast cancer was the most frequently diagnosed cancer in Saudi Arabia, with 4738 cases, followed by colorectal cancer, which ranked second with 3687 cases, and rectal cancer, which ranked fourth with 1899 cases. Liver and kidney cancers also appeared among the top 10 cancers, with 1432 and 1151 cases, respectively, ranking 8th and 10th. Liver cancer was responsible for 8.1% of all cancer deaths that year, followed by breast cancer at 7.5% and colorectal cancer at 6.9%^[4,104]^. Table 2 summarizes the obesity-linked cancers that are also among the most common in Saudi Arabia. Figure 4 presents the incidence and mortality figures for the top 10 cancers, several of which are obesity-related and account for a substantial share of cancer-related mortality. Figure 5 highlights the overlap between common cancers and those associated with obesity.

**Table 2:** Common obesity-linked cancers also among top cancers in Saudi Arabia (GLOBOCAN 2022)

Cancer type	Obesity link (WCRF/IARC)	Relative risk of the highest BMI category evaluated versus normal BMI (95% CI)	Rank in Saudi Cancer incidence (2022)	Rank in Saudi cancer deaths (2022)
Breast (postmenopausal)	Convincing	1.1 (1.1–1.2)	1st	2nd
Colon and rectum	Convincing	1.3 (1.3–1.4)	3rd, 4th	3rd, 5th
Liver	Convincing	1.8 (1.6–2.1)	8th	1st
Corpus uteri (Endometrial)	Convincing	7.1 (6.3–8.1)	9th	13th
Kidney (renal cell)	Convincing	1.8 (1.7–1.9)	10th	12th
Pancreas	Convincing	1.5 (1.2–1.8)	16th	9th
Ovary	Probable	1.1 (1.1–1.2)	19th	14^th^

Sources: WCRF/IARC evaluations and GLOBOCAN 2022 data.

**Figure 6.**
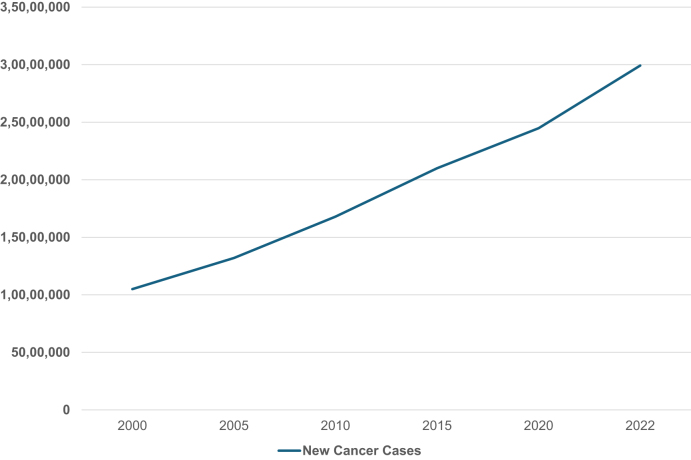
Rising trend in cancer incidence in Saudi Arabia (2000–2022). Total cancer incidence in Saudi Arabia has shown a consistent upward trend over the past two decades, reflecting an increasing national cancer burden.

The overlap between these cancer types in Saudi Arabia and those associated with obesity underscores the growing urgency of addressing the problem in the Kingdom. It is critical to recognize obesity as a primary modifiable risk factor. Figure 6 illustrates the consistent rise in overall cancer incidence in Saudi Arabia from 2000 to 2022, reflecting the broader impact of modifiable lifestyle factors such as obesity on national cancer trends.

## Current evidence from local studies

Although most Saudi studies have not primarily focused on obesity as the main exposure variable, several have reported significant correlations between elevated BMI or metabolic disorders and cancer occurrence. A few selected locally conducted investigations, summarized in Table 3, include breast, colorectal, thyroid, uterine, liver, and pancreatic malignancies and provide important contextual insights despite their limited scope. Collectively, they reveal that obesity and diabetes are highly prevalent among patients with cancer in Saudi Arabia and are frequently associated with more advanced disease stages or adverse tumor characteristics. However, the majority of available data are derived from single-center, retrospective, or cross-sectional studies, limiting causal interpretation. These findings, though preliminary, underscore the pressing need for prospective, population-based studies that systematically evaluate obesity, diabetes, and related metabolic parameters as independent cancer risk factors in the Saudi population

**Table 3:** Summary of Saudi Arabian studies examining the relationship between obesity, metabolic disorders, and cancer risk across different malignancies

Author (Year)	Study type/Design	Population & sample size	Cancer Type	Key findings	Limitations
Elkum N *et al* (2014)^[105]^	Unmatched case–control	1172 Arab women (534 BC cases, 638 controls)	Breast cancer	Obesity significantly increased breast cancer risk (OR = 2.29; 95% CI 1.68–3.13). Other significant predictors: family history, HRT, postmenopausal status, and lack of education.	Single-center case–control study; potential recall and selection bias; limited control for lifestyle confounders.
Alghamdi I *et al* (2015)^[106]^	Hospital-based case–control	675 Saudi women (225 cases, 450 controls)	Breast cancer	Early marriage (<18 years) was the strongest predictor (OR = 13.9); obesity (BMI ≥ 30 kg/m^2^) independently increased BC risk (OR = 5.7; 95% CI 2.53–13.0).	Hospital-based sample; limited adjustment for confounders; regional representation only.
Alshamsan B *et al* (2022)^[107]^	Retrospective cohort (registry-based)	2212 Saudi women with non-metastatic BC	Breast cancer	Obesity prevalence = 53.4%. Obesity associated with advanced stage, postmenopausal status, and triple-negative subtype among premenopausal women.	Retrospective design; incomplete long-term outcome data; single-country registry.
Alqahtani SM *et al* (2023)^[108]^	Retrospective observational	113 Saudi patients with AUS/FLUS thyroid nodules	Thyroid cancer	No significant correlation between BMI and malignancy (OR = 0.99; *P* = 0.87); higher malignancy in nonobese men (*P* = 0.04).	Small sample size; single-institution design; limited generalizability.
Alasiri G *et al* (2024)^[90]^	Cross-sectional analytical	319 Saudi colorectal cancer (CRC) patients (147 ♀, 172 ♂)	Colorectal cancer	57.9% of patients were overweight/obese; diabetes present in >60% of cases; significant CRC–diabetes association (OR = 1.44; *P* = 0.05).	Cross-sectional design; no control group; limited causal inference.
Alessa AM & Khan A (2024)^[109]^	Narrative review of national data	Summary of Saudi CRC epidemiology	Colorectal cancer	Identified modifiable risks – obesity (≈10% male, 7% female attributable risk), inactivity (OR = 8.5), and low education (OR = 8.3). Highlighted genetic risk variants (MMP2, GSTM1, XRCC1, VDR).	Narrative review; secondary data; potential publication bias; heterogeneous data sources.
Al-Kadri HM *et al* (2004)^[110]^	Retrospective observational	195 Saudi women with postmenopausal bleeding	Uterine (endometrial) cancer	Uterine cancer detected in 24.1% of cases; age > 60 years (OR 6.8–28.4) and ≥2 bleeding episodes (OR 4.5) significant; obesity, diabetes not significant predictors.	Historical cohort; small sample size; limited BMI and metabolic data.
Aldaqal SM *et al* (2020)^[111]^	Retrospective cross-sectional study	233 Saudi patients with histologically confirmed colorectal cancer	Colorectal cancer	Mean BMI = 26.5 kg/m^2^: 33% overweight, 30% obese. Adenocarcinoma most common (≈86%). No significant association between BMI category and age, tumor stage, histologic type, or nodal involvement (*P* > 0.05).	Single-center retrospective design; moderate sample size; lack of long-term survival data; limited multivariate analysis for confounders.
Alamri F, Saeedi M, Kassim K (2014)^[112]^	Case–control study	359 adults (174 CRC cases, 185 controls), age 20–85 years	Colorectal cancer	Mean BMI significantly higher in CRC cases (*P* = 0.03). Obesity associated with increased CRC risk (OR for obese = 1.6). Physical inactivity (OR = 3.21) and positive family history (OR = 1.23) were significant risk factors. NSAID use was protective (OR = 0.24, *P* < 0.001).	Hospital-based sample; modest size; potential recall bias; limited dietary quantification and confounder adjustment.
Ahmed AE *et al* (2018)^[113]^	Retrospective analytic study	206 patients who underwent pancreatic biopsy at a tertiary center in Saudi Arabia	Pancreatic cancer	57.8% of pancreatic biopsies were malignant. Diabetes was present in 56.8% of patients and was significantly more frequent in malignant cases. Older age, male gender, diabetes, weight loss, jaundice, and pancreatitis were independent predictors of malignancy.	Single-center retrospective design; limited generalizability; potential selection bias; unable to establish causality.
Coker T *et al* (2022)^[114]^	Population microsimulation model	Saudi adults (ages 20–59 years)	Liver disease & liver cancer	Predicted ≈2 million new T2DM cases and ≈2100 new liver cancer cases (2020–2040) attributable to obesity; projected healthcare cost ≈USD 128 billion.	Model-based analysis; dependent on input assumptions; no clinical verification.
Mahmoud MR *et al* (2025)^[115]^	Prospective comparative analytical	2748 adults (1242 Saudis; 1506 Egyptians)	Liver disease & hepatocellular carcinoma	Among Saudis, 35.5% had liver disease secondary to diabetes; fatty liver in 35.7%; obesity prevalence 63.8%. Confirmed bidirectional DM–liver disease relationship.	Multinational sample; observational design; limited longitudinal follow-up.

BC, breast cancer; HRT, hormone replacement therapy; AUS/FLUS, atypia of undetermined significance/follicular lesion of undetermined significance; NSAID, nonsteroidal anti-inflammatory drugs.

## Gaps in Saudi-specific studies

There is a well-documented global association between obesity and cancer, yet there remains a significant lack of local epidemiological research examining this relationship. Most available data on cancer incidence in the Kingdom do not include obesity-related metrics such as BMI, waist circumference, or metabolic profiles. As a result, there are limited data on how obesity contributes to the development, progression, and outcomes of cancer within the Saudi population. Furthermore, there are limited cohort or case–control studies in Saudi Arabia that are exploring the longitudinal relationship between obesity and specific cancer types. Most studies have treated obesity as a secondary variable or confounder rather than a primary risk factor. This limits the ability to generate high-quality evidence-based public health strategies or national screening policies tailored to high-risk populations. The absence of obesity-specific data in the Saudi Cancer Registry is also a factor. It provides valuable information on incidence and mortality, but it does not routinely capture anthropometric or lifestyle-related risk factors. This absence limits the development of predictive tools and risk stratification models that could otherwise improve early detection and prevention strategies.

**Figure 7.**
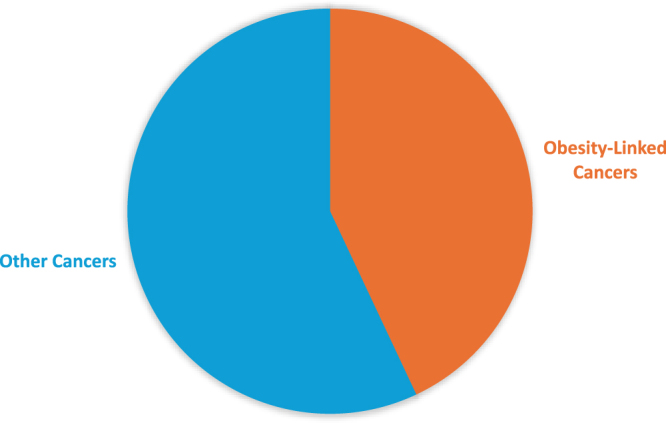
Proportion of obesity-linked cancers in Saudi Arabia’s total cancer burden (2022): Obesity-linked cancers, including breast, colorectal, liver, pancreas, prostate, and endometrial cancers, account for approximately 43% of total cancer cases in Saudi Arabia in 2022, highlighting the substantial impact of obesity as a modifiable cancer risk factor.

Even with well-established differences in obesity prevalence across Saudi regions and between men and women, there is minimal research evaluating regional and gender-based differences in obesity-related cancer risks. Table 4 outlines key research gaps that hinder the understanding and prevention of obesity-related cancers in the Saudi population. These challenges call for interdisciplinary cooperation among oncologists, epidemiologists, public health specialists, and policymakers. These measures will be crucial for developing focused cancer prevention initiatives, establishing national guidelines, and ultimately aiming to reduce the prevalence of obesity-related cancers in Saudi Arabia. Figure 7 illustrates that obesity-related cancers constitute about fifty percent of all cancer cases in Saudi Arabia, highlighting the critical necessity for targeted interventions.

**Table 4:** Research gaps in obesity-linked cancer epidemiology in Saudi Arabia

Gap area	Description	Suggested research action
Lack of local cohort studies	No long-term Saudi studies linking obesity to specific cancer risks	Establish prospective cohort studies with anthropometric tracking
Incomplete cancer registry data	National registry lacks BMI	Integrate obesity-related variables into Saudi Cancer Registry
Limited obesity–cancer stratification	Most studies do not stratify cancer outcomes by obesity status	Analyze survival and recurrence by obesity status in national data
Gender and regional disparities	Obesity patterns differ by sex and region but are not reflected in cancer data	Conduct region- and sex-specific cancer risk assessments
Absence of intervention trials	No trials evaluating impact of weight loss on cancer risk or outcomes	Launch community or hospital-based obesity prevention trials
Minimal public health surveillance	Lack of integration of obesity indicators in national NCD surveillance	Include cancer-related outcomes in obesity monitoring frameworks

## Policy implications for the Kingdom

The dual burden of high obesity prevalence and rising incidence of obesity-related cancers in Saudi Arabia calls for urgent policy action. Global evidence strongly supports the link between obesity and multiple cancer types, but the lack of Saudi-specific data limits the ability of healthcare policymakers to implement precise, evidence-based interventions. Public health policies in the Kingdom must begin to recognize obesity not only as a metabolic and cardiovascular risk factor but also as a cancer prevention priority. Incorporating obesity screening and weight management into routine primary care, especially for middle-aged and postmenopausal women, could have a significant impact on reducing breast, endometrial, and colorectal cancer incidence – cancers that dominate national cancer statistics^[4,116]^. The Vision 2030 Health Sector Transformation Program offers a strong framework for integrating obesity prevention with cancer control. This can include:

- Development of national guidelines on obesity screening for cancer risk stratification.
- Expansion of community-based physical activity programs targeting women and adolescents.
- Media campaigns highlighting the link between obesity and cancer.
- Enhanced nutrition education in schools and primary care settings.

Research funding must prioritize large-scale cohort studies. Upgrading the national cancer registry to include obesity-related data, such as BMI and metabolic markers at diagnosis, can help develop predictive models that will help in tailoring prevention strategies to regional obesity and cancer patterns^[21,23]^. Finally, training healthcare professionals to recognize obesity as a cancer risk factor is essential. This includes incorporating obesity–cancer education into continuing medical education programs for clinicians, particularly general practitioners and oncologists. Multidisciplinary approaches involving endocrinologists, nutritionists, and oncologists will be crucial for implementing effective, long-term strategies.

## Recommendations for future research

To effectively address the rising burden of obesity-related cancers in Saudi Arabia, future research efforts must focus on generating high-quality, locally relevant evidence. The following recommendations are proposed to guide researchers, institutions, and policy stakeholders:

1. Conduct Longitudinal Cohort Studies: Prospective studies are needed to investigate the long-term impact of obesity on cancer incidence and outcomes in the Saudi population. These should include detailed anthropometric measurements and metabolic parameters, stratified by age, sex, and region.
2. Integrate Obesity Data into National Cancer Registries: The Saudi Cancer Registry should be expanded to capture BMI, waist circumference, physical activity, and dietary patterns at the time of diagnosis. This would enable researchers to track obesity trends in relation to cancer incidence and survival outcomes.
3. Study Cancer Subtypes by Obesity Status: Research should examine how obesity influences tumor biology, treatment response, and prognosis for specific cancers common in Saudi Arabia, such as breast, colorectal, liver, and endometrial cancers.
4. Develop Obesity–Cancer Risk Prediction Models: Statistical models incorporating obesity metrics, family history, and metabolic markers could help identify high-risk individuals for targeted screening and early intervention.
5. Explore Regional and Gender Disparities: Studies should investigate how regional lifestyle patterns, cultural norms, and gender differences influence the relationship between obesity and cancer in the Saudi context.
6. Evaluate Public Health Interventions: Community-based trials are needed to assess the effectiveness of obesity prevention and weight loss programs in reducing cancer risk or improving outcomes in cancer survivors.
7. Promote Multidisciplinary Research Collaborations: National research funding agencies should support collaborations between universities, hospitals, public health departments, and global cancer research networks to foster knowledge exchange and capacity building.

These research priorities are essential for developing context-specific prevention strategies, improving early detection, and informing national cancer control policies that reflect the unique demographic and lifestyle characteristics of the Saudi population.

## Limitations

This review adopts a narrative rather than a systematic approach; therefore, the possibility of selection and interpretation bias cannot be fully excluded. To mitigate this limitation, we implemented a structured multi-database search strategy, applied predefined inclusion criteria, and prioritized high-quality, peer-reviewed studies. However, publication bias, heterogeneity among study designs, and the scarcity of population-based Saudi data may limit the comprehensiveness and generalizability of the conclusions. Future systematic reviews and longitudinal national studies are warranted to validate and expand upon these findings.

## Conclusion

Obesity is a rapidly growing global health concern, and Saudi Arabia stands among the countries most affected by this epidemic. The impact of obesity extends beyond metabolic and cardiovascular diseases, with a substantial and well-established association between excess body fat and an increased risk of various cancers. Alarming overlaps between the most common cancers in Saudi Arabia – such as breast, colorectal, liver, kidney, and endometrial cancers – and those known to be obesity-related signal a potential public health crisis that remains under-recognized. Despite the abundance of global evidence, there remains a significant research gap within Saudi Arabia regarding the direct link between obesity and cancer. Without national studies or registry data that explicitly track obesity-related cancer risk, health planners are left with limited tools to tackle a major modifiable determinant of cancer incidence and mortality.

Addressing this gap through targeted research, public health interventions, and policy-driven prevention strategies could have a transformative impact on cancer control efforts in the Kingdom. Integrating obesity prevention into cancer strategies, increasing public awareness, and implementing region-specific guidelines are essential next steps. As Saudi Arabia continues to modernize and reform its healthcare sector under Vision 2030, prioritizing obesity-related cancer prevention is not just a medical necessity – it is a strategic imperative to improve long-term population health outcomes.

## Data Availability

Not applicable.
